# Effect and Management of Excess Weight in the Context of Fertility-Sparing Treatments in Patients With Atypical Endometrial Hyperplasia and Endometrial Cancer: Eight-Year Experience of 227 Cases

**DOI:** 10.3389/fonc.2021.749881

**Published:** 2021-11-05

**Authors:** Ying Shan, Meng Qin, Jie Yin, Yan Cai, Yan Li, Yu Gu, Wei Wang, Yong-xue Wang, Jia-yu Chen, Ying Jin, Ling-ya Pan

**Affiliations:** ^1^ Department of Obstetrics and Gynecology, Peking Union Medical College Hospital, Chinese Academy of Medical Sciences and Peking Union Medical College, Beijing, China; ^2^ National Clinical Research Center for Obstetric & Gynecologic Diseases, Beijing, China

**Keywords:** fertility-sparing treatments, atypical endometrial hyperplasia (AEH), endometrial cancer (EC), excess weight, levonorgestrel intrauterine devices, gonadotropin-releasing hormone agonist (GnRH- a)

## Abstract

**Objective:**

To investigate the oncologic and reproductive outcomes of fertility-sparing treatments (FSTs) in atypical endometrial hyperplasia (AEH) and endometrial cancer (EC) patients with excess weight (EW).

**Methods:**

This retrospective study comprised patients with AEH or EC who achieved a complete response (CR) after FST from 2010 to 2018. The clinical characteristics, oncological and reproductive outcomes were compared between the excess weight (EW) group (body mass index (BMI)≥25 kg/m^2^) and normal weight (NW) group (BMI<25 kg/m^2^). The risk factors associated with recurrence and unsuccessful pregnancy in patients with EW were analyzed.

**Results:**

Overall, 227 patients were enrolled, including 139 (61.2%) in EW group and 88 (38.8%) in NW group. In patients with EW, the pregnancy rate, the live birth rate and the relapse rate were 29.8%, 23.4%, and 30.9%, respectively. In patients with NW, these rates were 61.1%, 47.2%, and 31.8%, respectively. No significant differences were observed in the time to remission (P=0.865) and disease-free survival (DFS) (P=0.750). Patients in NW group achieved a better pregnancy rate than patients in the EW group (P=0.034). The patients with EW using ovulation induction to increase fertility tended to have a shorter time to pregnancy (P=0.042). However, no significant risk factors associated with unsuccessful pregnancy were identified after the multivariate analysis. In terms of DFS, the combination of gonadotropin-releasing hormone agonist (GnRH-a) and LNG-IUD was better for patients with EW than GnRH-a or oral progestin therapy alone (P=0.044, adjusted hazard ratio (HR)=0.432, 95% confidence interval (CI): 0.152-1.229), especially for patients with EW diagnosed with EC (P=0.032).

**Conclusion:**

FSTs for overweight and obese patients should be more individualized. GnRH-a and/or LNG-IUD may be options prior to FSTs in patients with EW. Further prospective studies are needed.

## Introduction

Endometrial cancer (EC) is one of the most common malignant tumors in females (1). EC usually arises in postmenopausal women, but approximately 10% of EC patients are younger than 40 years old (2). Atypical endometrial hyperplasia (AEH) prior to EC represents a continuously changing disease process as AEH is a precancerous lesion of EC. The risk of AEH progressing to EC within fifteen years has been reported to be as high as 29.0% (3, 4). Thus, therapy for both AEH and EC should warrant attention.

In recent years, the incidence of young patients with AEH and EC has increased worldwide, and fertility-sparing treatments (FSTs) to preserve reproductive function are urgently needed (5). Approximately 80% of young EC patients have well-differentiated type I disease at a very early stage and high-estrogen exposure backgrounds, presenting the possibility of progestin-based therapy (6). The National Comprehensive Cancer Network (NCCN) has provided FST options for the management of AEH and EC patients who meet five specific criteria (7). The common recommended conservative treatments for AEH and EC include high-dose oral progestin and levonorgestrel intrauterine devices (LNG-IUDs). After FST, most AEH and EC patients can achieve a higher complete response (CR) rate and lower relapse rate and then successfully undergo delivery (8). Nevertheless, approximately one-quarter of females still suffer from recurrence problems. Gallos et al. showed that the relapse rates of patients with EC and AEH after FST were 40.6% and 26%, respectively, in a meta-analysis of 34 observational studies (9). The curative effect and prognostic outcomes after recurrence were not satisfactory. Therefore, the risk factors for recurrence must be identified to decrease the risk of recurrence.

The relationship between obesity and endometrial cancer (EC) has been established and accepted for decades. Overweight and obesity are evaluated by body mass index (BMI), which is a measurement of a person’s weight with respect to his or her height. The World Health Organization (WHO) defines an adult who has a BMI between 25 kg/m^2^ and 29.9 kg/m^2^ as overweight and an adult who has a BMI of 30 kg/m^2^ or higher as obese (10). On the one hand, the risk of EC increases with increasing weight according to several previous reports (11). Compared with normal-weight women, the relative risk (RR) and odds ratio for developing EC were 1.34 and 1.43 in overweight women and 2.54 and 3.33 in obese women, respectively (12). The previous study from our single team reported that age ≥35 years, obesity, prolonged time to CR, and consistent infertility after conservative treatment were associated with an increased risk of recurrence (9). In addition, although the mortality rate of EC is low, the RR of death is significantly higher for obese EC patients than for those with a normal BMI (RR 2.53 for BMI 30–34 kg/m^2^, RR 6.25 for BMI > 40 kg/m^2^) (13). Thus, we can solve most of treatment and recurrence problems related to FSTs if we can solve the treatment problems encountered in obese patients with AEH and EC. On the other hand, FSTs for obese patients are challenging. Obese patients with EC often have multiple complications, such as polycystic ovarian syndrome (PCOS), diabetes mellitus (DM), and hypertension. Moreover, obesity not only is a risk factor for developing EC but may also significantly impact pregnancy (14). High-dose oral progestin, as the most common FST, has side effects, including weight gain, abnormal lipid metabolism, and compromised liver function. These side effects limit its application in overweight patients and may lead to an increased risk of recurrence (15). Recently, the use of gonadotropin-releasing hormone agonist (GnRH-a) has become widely popularized for obese patients in clinical practice, but NCCN guidelines do not provide specific treatment recommendations (16).

Therefore, we performed this retrospective study to explore the oncologic and reproductive results of FSTs in patients with excess weight (EW) with AEH and EC, as well as risk factors for unsuccessful and recurrent pregnancy. Developing more suitable management strategies is important for such populations.

## Materials And Methods

### Study Population

This retrospective study included all patients with AEH and EC who received FSTs between January 2010 and December 2018 at Peking Union Medical College Hospital (PUMCH). Patients with EW were defined as having a BMI equal to or greater than 25 kg/m^2^. In our institution, patients were considered candidates for FST when they met the following criteria, which were almost consistent with NCCN guidelines (7): 1) age younger than 40 years old and a strong desire for fertility preservation; 2) a diagnosis of EC of a well-differentiated type (G1) or AEH through dilation and curettage (D&C) with or without hysteroscopy, with confirmation of the pathological diagnosis by at least two experienced gynecological pathologists; 3) a tumor confined to the endometrium with no evidence of myometrial invasion as evaluated by transvaginal ultrasonography and pelvic magnetic resonance imaging (MRI); 4) Estrogen receptor (ER) and progesterone receptor (PR) positivity; 5) a normal serum CA125 level; 6) no contraindications for progestin therapy or other medical therapy; 7) fertility function assessment prior to FST; and 8) an understanding through counseling that fertility-sparing option is not a standard of care for the treatment of EC and provision of written informed consent. This study was conducted with the approval of the Ethics Committee of PUMCH.

### Data Collection

All included patients were provided counseling regarding their FST options, including the side effects of the drugs and potential risks of recurrence or progression. Patients who met the study inclusion criteria were divided into two groups: the excess weight (EW) group, BMI of which was equal to or more than 25 kg/m^2^; the normal weight (NW) group, BMI of which was less than 25 kg/m^2^. In this study, the treatment methods were divided into four groups: 1) MPA; 2) MA; 3) GnRH-a; and 4) GnRH-a+LNG-IUD. Oral progestin therapy is one of the most common primary FSTs and includes medroxyprogesterone acetate (MPA) at doses of 250-500 mg/day or megestrol acetate (MA) at doses of 160-480 mg/day. If a patient with EC or with extended lesion, GnRH-a was administered for three to six cycles (3.6 mg/3.75 mg) by subcutaneous injection as the first treatment according to experience. In addition, an LNG-IUD could be placed in combination for treatment. The patients using common therapy in combination with letrozole or only LNG-IUD were in a small number, which were excluded from this study to avoid results bias. The response to treatment was assessed every 3-6 months using pathological specimens, which were obtained *via* D&C and hysteroscopy. CR was defined as the absence of endometrial hyperplasia or carcinoma. Patients were recommended to receive regular maintenance treatment after CR while waiting for fertility or not having fertility willing, which included LNG-IUD or low-dose oral progestin. The ultrasound should be administered every 3 to 6 months during maintenance therapy.

All willing patients with immediate fertility after a CR were transferred to the specialized reproductive center to undergo counseling regarding reproductive treatment options. In a general way, the patients with a preferable ovarian reserve and successful ovulation, as well as smooth fallopian tubes, were encouraged to conceive spontaneously. Patients with anovulation were recommended track their sex life and induce ovulation with letrozole, which was administered at a dose of 2.5 mg/day for 5 days. For patients with a reduced ovarian reserve, anovulation, or PCOS, assisted reproductive technology (ART) was encouraged as early as possible. These methods included intrauterine insemination (IUI) or *in vitro* fertilization and embryo transfer (IVF-ET). The time to pregnancy was defined as the time interval between the date that CR was achieved and the date that a pregnancy was confirmed by ultrasound examination or the final follow-up.

Recurrence was defined as initial lesions (AEH or EC) reappearing in the specimen after complete remission or a disease lesion reappearing in the endometrium and/or myometrium on imaging examination. Patients with EW were divided into two groups: the recurrence group and the control group. Disease-free survival (DFS) was defined as the time interval between the date that CR was achieved and the date of recurrence or the final follow-up. For this study, patients with any of the following characteristics were excluded: 1) received other treatments except MA, MPA and GnRHa ± LNG-IUD; 2) did not achieve CR after FST; 3) were not regularly evaluated every three months *via* D&C or hysteroscopy during the treatment period; and 4) were not followed up regularly after CR at PUMCH or a local hospital.

### Statistical Analysis

All statistical analyses were performed using SPSS software (version 23.0; SPSS Inc., Chicago, IL, USA), and graphs were generated using GraphPad Prism software for Macbook (version 7.0; GraphPad software Inc., San Diego, USA). Student’s t-tests and Mann-Whitney U tests were used to compare continuous variables. Pearson’s chi-squared tests and Fisher’s exact tests were used to compare categorical variables (17). Survival analysis was performed using Kaplan-Meier curves and the log-rank test. Each factor related to survival outcomes was individually evaluated using a Cox regression model in a univariate analysis. Then, all variables with *P* values <0.200 and meaningful variables based on the univariate analysis were included in the Cox proportional hazards regression model in a multivariate analysis. The associations of these variables with follow-up outcomes was evaluated by hazard ratios (HRs) and 95% confidence intervals (CIs). Statistical significance was set at *P*<0.050.

## Results

### The Clinical Characteristics, Treatment Choices, and Follow-up Outcomes of the Included Patients

**Table 1** shows the clinical and pathological characteristics of AEH and EC patients after FST between EW and the NW group. Overall, 227 patients who met the inclusion criteria were included in this retrospective analysis. The NW group contained 88 (38.8%) patients, while the EW group contained 139 (61.2%) patients, including 74 (53.2%) patients with a 25≤BMI<30 kg/m^2^ and 65 (46.8%) patients with a BMI≥30 kg/m^2^. The mean BMI of all patients was 26.8( ± 5.2) kg/m^2^ (ranging from 18.0 to 46.5 kg/m^2^), and the mean age was 31.5 ( ± 4.7) years. A total of 29.1% of patients had complications, including PCOS (20.7%), DM (5.3%), and others (3.1%). A total of 63.0% of patients were diagnosed with AEH, and 37.0% were diagnosed with EC. The median follow-up time was 41.7 ( ± 23.0) months. No significant differences were observed in complications (*P*=0.120), menstruation cycle (*P*=0.190), previous pregnancy (*P*=0.050), previous delivery (*P*=0.063), and histology (*P*=0.902). Therefore, most of variables were equally comparable for the survival analysis.

**Table 1:** The clinical characteristics of AEH and EC patients after FST between the excess weight group and normal weight group.

Variable	Total (N = 227)	Excess weight group (N = 139)	Normal weight group (N = 88)	*P*
**BMI (kg/m^2^)**				
Mean ± SD	26.8 ± 5.2	30.1 ± 3.9	21.7 ± 1.8	
Range	18.0-46.5	25.0-46.5	18.0-24.8	
**Age (year)**	31.5 ± 4.7	32.2 ± 4.5	30.4 ± 4.8	0.028
≤35	182 (80.2%)	105 (75.5%)	77 (87.5%)	
>35	45 (19.8%)	34 (24.5%)	11 (12.5%)	
**Complications**				0.120
No	161 (70.9%)	91 (65.5%)	70 (79.5%)	
PCOS	47 (20.7%)	33 (23.7%)	14 (15.9%)	
Diabetes mellitus	12 (5.3%)	9 (6.5%)	3 (3.5%)	
Others	7 (3.1%)	6 (4.3%)	1 (1.1%)	
**Menstruation cycle**				0.190
Regular	114 (50.2%)	65 (46.8%)	49 (55.7%)	
Irregular	113 (49.8%)	74 (53.2%)	39 (44.3%)	
**Previous pregnancy**				0.050
No	150 (66.1%)	85 (61.2%)	65 (73.9%)	
Yes	77 (33.9%)	54 (38.8%)	23 (26.1%)	
**Previous delivery**				0.063
No	182 (80.2%)	106 (76.3%)	76 (86.4%)	
Yes	45 (19.8%)	33 (23.7%)	12 (13.6%)	
**Histology**				0.902
AEH	143 (63.0%)	88 (63.3%)	55 (62.5%)	
EC	84 (37.0%)	51 (36.7%)	33 (37.5%)	
**Follow-up time (Months)**	41.7 ± 23.0	39.7 ± 21.5	44.8 ± 25.0	

Data are presented as number (%) or mean ( ± SD) or median ( ± IQR). BMI, body mass index; PCOS, polycystic ovary syndrome; AEH, atypical endometrial hyperplasia; EC, endometrial cancer.

**Figure 1:**
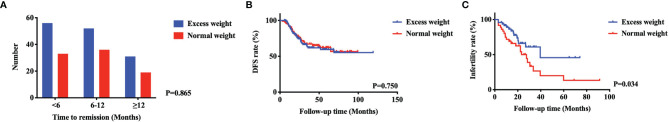
The comparison of AEH and EC patients after FST between excess weight group and normal weight group. There were no significant differences of the time to remission **(A)** and DFS **(B)** between two groups. The patients in NW group had better pregnancy rate than in EW group **(C)**.

**Figure 2:**
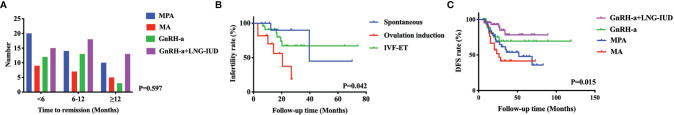
The risk factors associated with infertility for AEH and EC patients of excess weight with pregnancy intention. The time to remission among the four treatment types did not significantly differ in patients with EW **(A)**. In the univariate analysis, the patients with EW who used ovulation induction to promote conception had the shortest time to pregnancy, followed by those who used IVF-ET and those who achieved spontaneous pregnancy **(B)**. The patients with EW treated with GnRH-a+LNG-IUD had the best DFS, followed by those treated with GnRH-a and MPA, and the worst DFS was observed in patients treated with MA **(C)**.

The treatment, follow-up and reproductive outcomes of the included patients are shown in **Table 2**. The treatment methods were divided into four groups: MPA (40.1%), MA (17.2%), GnRH-a (17.6%), and GnRH-a+LNG-IUD (25.1%). A total of 51.1% of patients received regular maintenance treatment, including LNG-IUDs (73.3%) and Duphaston (26.7%). The mean time to a CR was 8.7 ( ± 5.6) months, and 22% patients need more than 12 months. The time to remission among the four treatment types did not significantly differ (*P*=0.597) in patients with EW, as shown in **Figure 2A**. A total of 33.8% of patients with EW had an immediate pregnancy intention after remission and attempted to become pregnant by different methods. Among these patients with EW, 27.7% of patients spontaneously became pregnant, 23.4% used ovulation induction, and the others used IVF-ET to improve their chances of conceiving. In patients with EW, the pregnancy rate, the live birth rate and the relapse rate were respectively 29.8%, 23.4%, and 30.9%. While in patients with NW, the pregnancy rate, the live birth rate and the relapse rate were respectively 61.1%, 47.2%, and 31.8%. There were no significant differences of the time to remission between EW group and NW group in **Figure 1A** (*P*=0.865). The patients in NW group showed similar DFS with patients in EW group in **Figure 1B** (*P*=0.750, HR=0.926, 95% CI: 0.577-1.485). However, the patients in NW group had better pregnancy rate than in EW group (*P*=0.034, HR=2.023, 95% CI: 1.047-3.909), as shown in **Figure 1C**.

**Table 2:** The treatment, follow-up and reproductive outcomes of AEH and EC patients after FST between the excess weight group and normal weight group.

Variable	Total (N = 227)	Excess weight group (N = 139)	Normal weight group (N = 388)	*P*
**Treatment**				0.001
MPA	91 (40.1%)	44 (31.7.%)	47 (53.4%)	
MA	39 (17.2%)	21 (15.1%)	18 (20.5%)	
GnRH-a	40 (17.6%)	28 (20.1%)	12 (13.6%)	
GnRH-a+LNG-IUD	57 (25.1%)	46 (33.1%)	11 (12.5%)	
**Time to CR (Months)**	8.7 ± 5.6	8.6 ± 5.6	8.8 ± 5.6	0.865
<6	89 (39.2%)	56 (40.3%)	33 (37.5%)	
≥6, <12	88 (38.8%)	52 (37.4%)	36 (40.9%)	
≥12	50 (22.0%)	31 (22.3%)	19 (21.6%)	
**Regular maintenance treatment**				0.993
No	111 (48.9%)	68 (48.9%)	43 (48.9%)	
Yes	116 (51.1%)	71 (51.1%)	45 (51.1%)	
LNG-IUDs	85 (73.3%)	53 (74.6%)	32 (71.1%)	
Duphaston	31 (26.7%)	18 (25.4%)	13 (28.9%)	
**Pregnancy Method**				
without pregnancy intention	144 (63.4%)	92 (66.2%)	52 (59.1%)	0.279
with pregnancy intention	83 (36.6%)	47 (33.8%)	36 (40.9%)	0.084
spontaneous	24 (29.0%)	13 (27.7%)	11 (30.6%)	
ovulation induction	19 (22.9%)	11 (23.4%)	8 (22.2%)	
IVF-ET	40 (48.1%)	23 (48.9%)	17 (47.2%)	
**Pregnancy outcome**				
without pregnancy intention	144 (63.4%)	92 (66.2%)	52 (59.1%)	0.279
with pregnancy intention	83 (36.6%)	47 (33.8%)	36 (40.9%)	0.017
Live birth	28 (33.7%)	11 (23.4%)	17 (47.2%)	
Spontaneous abortion	8 (9.6%)	3 (6.4%)	5 (13.9%)	
Non-pregnant	47 (56.7%)	33 (70.2%)	14 (38.9%)	
**Recurrence**				0.889
No	156 (68.7%)	96 (69.1%)	60 (68.2%)	
Yes	71 (31.3%)	43 (30.9%)	28 (31.8%)	
**Recurrent histology**				0.617
AEH	54 (76.1%)	31 (72.1%)	23 (82.1%)	
EC	17 (23.9%)	12 (27.9%)	5 (17.9%)	

Data are presented as number (%). MPA, Medroxyprogesterone; MA, Megestrol acetate; LNG-IUD, levonorgestrel intrauterine device; GnRH-a, Gonadotropin releasing hormone agonist; CR; complete remission; AEH, atypical endometrial hyperplasia; EC, endometrial cancer; IVF-EF, In vitro fertilization and embryo transfer.

### Risk Factors Associated With Unsuccessful Pregnancy Among the Included Patients With EW

The risk factors associated with unsuccessful pregnancy among AEH and EC patients with EW after FSTs with pregnancy intention were further analyzed. In the univariate analysis shown in **Table 3**, the *P* values of the following two factors were less than 0.050: histology (*P*=0.009), and pregnancy method (*P*=0.042). In **Figure 2B**, the patients with EW who used letrozole for ovulation induction to promote conception had the shortest time to pregnancy, followed by those who used IVF-ET and those who achieved spontaneous pregnancy. However, there were no significant risk factors associated with unsuccessful pregnancy after the multivariate analysis.

**Table 3:** The univariate analysis and multivariate analysis of risk factors associated with infertility for AEH and EC patients of excess weight after FST with pregnancy intention.

Variables	N	Univariate analysis			Multivariate analysis		
		HR	95% CI	*Pp*	HR	95%CI	*P*
**Age (year)**				0.089			
≤35	35	1					
>35	12	0.171	0.022-1.313				
**Complications**				0.964			
No	32	1					
PCOS	10	0.681 0.227	0.147-3.148				
Diabetes mellitus	2	1.057	0.130-8.580				
Others	3	1.106	0.140-8.711				
**Menstruation cycle**				0.161			
Regular	21	1					
Irregular	26	2.294	0.718-7.331				
**Previous pregnancy**				0.367			
No	30	1					
Yes	17	0.579	0.176 -1.899				
**Histology**				0.009			0.052
AEH	29	1			1		
EC	18	4.385	1.445-13.306		3.270	0.992-10.780	
**Regular maintenance treatment**				0.662			
No	19	1					
Yes	28	1.296	0.405-4.151				
**Treatment**				0.714			
MPA	11	1					
MA	10	1.165	0.258-5.270				
GnRH-a	11	1.304	0.282-6.027				
GnRH-a +LNG-IUD	15	0.559	0.112-2.789				
**Recurrence**				0.098			
No	26	1					
Yes	21	2.568	0.841-7.840				
**Pregnancy**				0.042			0.229
spontaneous	13	1			1		
ovulation induction	11	5.696	1.092-29.722		3.020	0.518-17.599	
IVF-ET	23	1.623	0.324-8.139		1.137	0.217-5.967	

PCOS, polycystic ovary syndrome; AEH, atypical endometrial hyperplasia; EC, endometrial cancer; MPA, Medroxyprogesterone; MA, Megestrol acetate; LNG-IUD, levonorgestrel intrauterine device; GnRH-a, Gonadotropin releasing hormone agonist; IVF-EF, In vitro fertilization and embryo transfer.

Similarly, in the subgroup analysis of initial pathology, the oncologic outcomes of the included patients with EW characterized by the pregnancy method were further analyzed. There were no significant differences among three pregnancy methods in AEH (P=0.456) and EC (P=0.111) patients with EW, which were respectively shown in **Figures S1A, B**.

### Risk Factors Associated With Recurrence Among the Included Patients With EW

We analyzed the risk factors associated with recurrence for AEH and EC patients with EW after FSTs. In the univariate analysis, as shown in **Table 4**, the *P* values of the following two factors were less than 0.050: the absence of regular maintenance treatment (*P*=0.017) and treatment type (*P*=0.015). In **Figure 2C**, the patients with EW treated with GnRH-a+LNG-IUD had the best DFS (HR=0.309, 95%CI: 0.122-0.778), followed by those treated with GnRH-a and MPA, and the worst DFS was observed in patients treated with MA. After multivariate analysis, the treatment type was the risk factors associated with recurrence for AEH and EC patients with EW (*P*=0.044, adjusted HR=0.432, 95% CI: 0.152-1.229).

**Table 4:** The univariate analysis and multivariate analysis of risk factors associated with recurrence for AEH and EC patients of excess weight after FST.

Variables	N	Univariate analysis			Multivariate analysis		
		HR	95% CI	*Pp*	HR	95%CI	*P*
**Age (year)**				0.380			
≤35	105	1					
>35	34	1.349	0.691-2.632				
**Complications**				0.111			
No	91	1					
PCOS	33	0.306 0.	0.109-0.863				
Diabetes mellitus	9	0.442	0.106-1.842				
Others	6	0.703	0.169-2.930				
**Menstruation cycle**				0.097			
Regular	65	1					
Irregular	74	1.737	0.905-3.333				
**Previous pregnancy**				0.362			
No	85	1					
Yes	54	1.322	0.725-2.412				
**Histology**				0.089			
AEH	88	1					
EC	51	0.552	0.278-1.095				
**Regular maintenance treatment**				0.017			0.181
No	68	1			1		
Yes	71	0.474	0.257-0.877		0.614	0.300-1.255	
**Treatment**				0.015			0.044
MPA	44	1			1		
MA	21	1.416	0.681-2.943		1.730	0.790-3.789	
GnRH-a	28	0.613	0.256-1.470		0.755	0.301-1.894	
GnRH-a+LNG-IUD	46	0.309	0.122-0.778		0.432	0.152-1.229	

PCOS, polycystic ovary syndrome; AEH, atypical endometrial hyperplasia; EC, endometrial cancer; MPA, Medroxyprogesterone; MA, Megestrol acetate; LNG-IUD, levonorgestrel intrauterine device; GnRH-a, Gonadotropin releasing hormone agonist.

In the subgroup analysis of initial pathology, we further analyzed the oncologic outcomes of the included patients with EW characterized by treatment method. Similar tendencies for oncologic outcomes among EC patients with EW (*P*=0.032) are shown in **Figure S1D**. However, there was no significant difference among four treatment methods in AEH patients with EW (P=0.289), as shown in **Figure S1C**.

## Discussion

BMI plays an important role in the occurrence and development of AEH and EC. Approximately two-thirds of EC patients are obese, and the risk of EC is 2- to 5-times higher among obese women, which can be explained by the fact that increased sex hormone production from adipose tissue causes unopposed estrogen stimulation in the endometrial lining, similar to what occurs in breast cancer (6). Our previous study reported that obesity is not a risk factor for recurrence in AEH and EC patients after FST, although EW led to a higher incidence rate of EC and AEH (9). Gonthier et al. also showed that obese patients with AEH and EC also had similar CR rates and relapse rates compared with nonobese patients (18). However, a study performed by von Greunigen and the Gynecologic Oncology Group (GOG) showed that obesity increases the risk of mortality among women with a diagnosis of EC (19). In addition, obese patients often also have insulin resistance or cardiovascular diseases, which may lead to a longer therapeutic duration and poor prognosis for AEH and EC patients after FST (20). Therefore, overweight and obesity are nonnegligible risk factors during therapy for AEH and EC.

For AEH or EC patients without any contraindications receiving FSTs, the common treatment is high-dose progestin (21). However, the most common side effect of high-dose oral progestin is an increase in BMI, which creates a very large challenge for overweight patients trying to control their body weight (22). In addition, high-dose oral progestin may lead to elevated liver enzymes, which is also a very unfavorable condition for obese patients, especially those with DM or hypertension (23). Cholakian et al. showed that in patients with a BMI ≥ 35 kg/m^2^, MA was associated with more weight gain than LNG-IUDs (+2.2 *vs* -5.40 kg, *P*=0.05) (24). Regarding conservative treatment for EC, weight change is one of the evaluation indices for treatment effect and prognostic outcomes (25). Park et al. reported that a BMI≥25 kg/m^2^ before and after treatment is an important predictor for poor treatment response and high recurrence rate (25). The treatments for obesity include diet control, exercise, drugs, and bariatric surgery (26). The NCCN guidelines have indicated the importance of effective weight control and healthy lifestyle management for overweight patients. In our institution, overweight patients are also required to seek advice for weight loss in the nutrition department while undergoing FSTs. It is essential for patients to maintain a normal BMI during progestin treatment. Therefore, oral progestin may not be an optimal option for overweight AEH and EC patients, as the most common side effect is weight gain (22).

GnRH-a and LNG-IUDs as effective and acceptable forms of treatment have been used for multiple purposes by thousands of women worldwide. In our study, the combined use of GnRH-a and LNG-IUDs yielded the best DFS trend among treatments, especially for overweight EC patients. On the one hand, this result can be explained by a lower probability of weight gain with an GnRH-a and LNG-IUD. Cholakian et al. reported that the median weight change during therapy was greater with MA than with LNG-IUDs (+2.95 *vs.* +0.05 kg, *P*=0.03) (24). On the other hand, LNG-IUDs played an important role in maintaining endometrial thinning. Additionally, GnRH-a can inhibit the hypothalamic pituitary-gonadal axis in the central nervous system. Thus, the combination of LNG-IUDs and GnRH-a was a comparably effective method to suppress the production of estrogen from both the ovaries and peripheral tissue. Other studies have reported results similar to those of our study. A systematic review of 19 articles showed that LNG-IUDs had an advantage over oral progestin (27). Women with AEH were more likely to show regression with an LNG-IUD than with oral progestin. Furthermore, GnRH is an effective fertility-sparing strategy for women with AEH and EC due to the low recurrence rate of these diseases and the absence of progressive disease; this method achieved a good long-term uterine preservation rate and a high pregnancy rate (16, 28). Therefore, the abovementioned studies and our study provide evidence that LNG-IUDs and GnRH-a are preferred in the treatment of overweight and obese patients with AEH and EC (16).

On the one hand, letrozole belongs to a class of medications known as aromatase inhibitors and acts by blocking estrogen production and causing the pituitary gland to increase its stimulation of ovarian follicles (29). NCCN guidelines have recommended that letrozole can be used to enhance ovulation as the first-line treatment (7). Additionally, letrozole has been reported to be an effective ovulation induction agent in higher-BMI women (30). Obese patients usually have PCOS or ovulation disorders, which are important risk factors for infertility. Insulin resistance caused by obesity may exacerbate hyperandrogenism, and hyperandrogenism can increase the resistance to insulin, thus forming a vicious cycle (31). Letrozole can inhibit the growth of nondominant follicles and promote the development of single follicles (32). Letrozole has a short drug half-life and is thought to cause fewer antiestrogenic side effects on estrogen target organs than clomiphene citrate because ERs are not directly affected (33). In summary, letrozole can increase the sensitivity of patients to gonadotropin and improve impaired ovarian function due to obesity (34, 35). In our study, overweight patients using letrozole for ovulation induction to promote conception may have improved pregnancy outcomes to some extent, while IVF-ET was not an optimal choice. This can be explained by the fact that most patients using IVF-ET may indeed have infertility syndromes, and the successful pregnancy rate was originally lower. According to guidelines recommendations, obese patients should not undergo IVF until their BMI drops to below 30 kg/m^2^. On the other hand, letrozole can be used for FST in patients with AEH and EC from European Society of Gynecological Oncology (ESGO) guideline (36). This kind of treatment has been reported in our institution, as well as in some literatures (37–40). A previous study reported that the combination of GnRH-a and aromatase inhibitors showed a beneficial long-term outcome in young obese EC patients who wished to receive FST (40). However, the use of letrozole for FST was in a small number, and not more high evidence-based studies supported. Thus, in our institution, letrozole was mainly applied to induce ovulation in most overweight patients.

The results of our study showed that for patients with EW, maintenance treatment tend to reduce the recurrence rate of AEH and EC and increase the likelihood of maintaining regular menstruation, regardless of pregnancy intention after CR (21). The main maintenance treatments in this study were LNG-IUDs or low-dose oral progestin. Our previous studies have confirmed that regular oral progestin also significantly prolonged the DFS (RR=4.726; 95% CI: 2.672– 8.359) of young patients with AEH and EC (41). Wang et al. also showed that maintenance therapy was an independent protective factor for recurrence (*P*=0.001), while DM was an independent risk factor for recurrence (*P*=0.003) (42). Park et al. reported that if patients want to maintain fertility after childbirth, they can choose to use periodic oral contraceptives or LNG-IUDs to prevent recurrence (43). Therefore, it is considerable to use regular maintenance treatment after remission, regardless of whether the patients are considered normal weight or excess weight.

Nonetheless, there are several limitations in this study. First, unknown potential confounders and selection biases may be present in this retrospective institutional study due to the long period of data collection. However, we attempted to define the patient inclusion criteria carefully to ensure that all data were collected in a similar way and to always ensure uniformity between the two groups stratified by BMI. Moreover, we balanced confounding factors in the EW group with a Cox multivariate regression analysis when heterogeneities were present in the baseline factors. We also divided the dataset into homogenous subgroups and performed a stratification analysis. Second, the conservative treatments for AEH and EC patients are relatively unique. There has been no uniform standard for FST until now. This study retrospectively summarized the characteristics of existing cases and proposed guidelines. In clinical practice, gynecologic oncologists should pay more attention to the weight loss of overweight patients and adopt more personalized treatment options for such patients (11). For obese patients who have no histological response to the primary therapy for over 6-12 months, an alternative therapy strategy should be actively applied. Overweight patients should be informed that they may have an elevated risk of failed conservative treatment (44). In the future, we will continue to analyze more AEH and EC patients after FSTs and collect more information from new patients.

## Conclusion

In conclusion, most patients with AEH and EC who undergo FSTs are overweight and obese. The combination of GnRH-a and LNG-IUD produced better outcomes in patients with EW than GnRH-a or oral progestin therapy alone, especially for patients with EW diagnosed with EC. GnRH-a and/or LNG-IUD may be options prior to FSTs in patients with EW due to the low relapse rates of AEC and EC. Furthermore, patients with EW using ovulation induction to boost fertility tend to have a shorter time to pregnancy. The use of regular maintenance treatment after remission is recommended. Fertility-sparing management should not necessarily be contraindicated in overweight and obese patients, but the therapy and reproductive strategy should be more individualized. Further prospective studies are needed to investigate the underlying factors associated with oncologic and pregnancy outcomes.

## Data Availability Statement

The raw data supporting the conclusions of this article will be made available by the authors, without undue reservation.

## Ethics Statement

This study was conducted with the approval of the Ethics Committee of Peking Union Medical College Hospital. The patients/participants provided their written informed consent to participate in this study.

## Author Contributions

Study concepts: L-yP, and YJ. Study design: YS, MQ, L-yP, and YJ. Data acquisition: YS, MQ, YG, and WW. Quality control of data and algorithms: JY, Y-yC, and YL. Data analysis and interpretation: YS, MQ, and Y-xW. Statistical analysis: YS, MQ, and YC. Manuscript preparation: YS and MQ. Manuscript editing: All authors. Manuscript review: L-yP, YJ, and JY. All authors contributed to the article and approved the submitted version.

## Funding

This project was supported by The Fund of The National Key R&D Program of China 2016YFC1303700 (Affiliated project 2016YFC1303701, 2016.9-2020.12). Furthermore, this project was also supported by CAMS Innovation Fund for Medical Sciences (CIFMS-2017-I2M-1-002, 2017.01-2020.12). Besides, MQ was supported by China Scholarship Council (201906210463).

## Conflict of Interest

The authors declare that the research was conducted in the absence of any commercial or financial relationships that could be construed as a potential conflict of interest.

## Publisher’s Note

All claims expressed in this article are solely those of the authors and do not necessarily represent those of their affiliated organizations, or those of the publisher, the editors and the reviewers. Any product that may be evaluated in this article, or claim that may be made by its manufacturer, is not guaranteed or endorsed by the publisher.
