# Supplementary material for: Effect and Management of Excess Weight in the Context of Fertility-Sparing Treatments in Patients With Atypical Endometrial Hyperplasia and Endometrial Cancer: Eight-Year Experience of 227 Cases
Source: Front Oncol. 2021 Nov 5;11:749881. doi: 10.3389/fonc.2021.749881 (PMC8602817; doi:10.3389/fonc.2021.749881)
Supplement: Supplementary file 1 [file DataSheet_1.docx]

**Supplementary materials**

**Figure S1. The risk factors associated with infertility and recurrence for AEH and EC patients of excess weight with pregnancy intention.**

There were no significant differences among three pregnancy methods, respectively in AEH (A) and EC (B) patients with EW. There was no significant difference among four treatment methods in AEH patients with EW (C). The patients with EW treated with GnRH-a+LNG-IUD had the best DFS, followed by those treated with GnRH-a and MPA, and the worst DFS was observed in patients treated with MA (D).
